# Intermediate outcome for the single-stage surgical repair of aortopulmonary window associated with interrupted aortic arch

**DOI:** 10.1093/icvts/ivad077

**Published:** 2023-05-15

**Authors:** Chi Hoang Linh Nguyen, Truong Ly Thinh Nguyen, Vinh Quang Tran, Mai Tuan Nguyen, Duyen Dinh Mai, Anh Vuong Doan, Quang Ngoc Nguyen

**Affiliations:** Department of Cardiology, Hanoi Medical University, Hanoi, Vietnam; Department of Cardiovascular Surgery, Children's Heart Center, National Children’s Hospital, Hanoi, Vietnam; Department of Cardiovascular Surgery, Children's Heart Center, National Children’s Hospital, Hanoi, Vietnam; Department of Cardiovascular Surgery, Children's Heart Center, National Children’s Hospital, Hanoi, Vietnam; Department of Cardiovascular Surgery, Children's Heart Center, National Children’s Hospital, Hanoi, Vietnam; Department of Cardiovascular Surgery, Children's Heart Center, National Children’s Hospital, Hanoi, Vietnam; Department of Cardiology, Hanoi Medical University, Hanoi, Vietnam

**Keywords:** interrupted aortic arch, aortopulmonary window

## Abstract

**OBJECTIVES:**

Aortopulmonary window (APW) associated with an interrupted aortic arch (IAA) is a rare cardiac malformation with significant mortality and morbidity. The goal of this study was to report the intermediate outcomes of single-stage repair concentrating on the surgical techniques and postoperative reintervention for this rare cardiac lesion.

**METHODS:**

Eleven patients were diagnosed with IAA-associated APW and underwent single-stage surgical repair at Vietnam National Children’s Hospital.

**RESULTS:**

The APW anatomy types were types I, II and III in 1, 4 and 6 patients, respectively. The IAA morphology was type A in 9 patients and type B in 2 patients. The median age was 27 [interquartile range (IQR) 6–79] days, and the median weight was 3.5 (IQR 2.8–4.5) kg. The aortic arch was repaired using direct end-to-side tissue anastomosis in 7 patients, and patch aortoplasty was performed in 4 patients. Six patients underwent APW closure with an intra-aortic baffle, and 5 patients required right pulmonary artery detachment and reimplantation. One early death occurred. Four patients required reinterventions: 1 patient required reoperation due to aortic stenosis and 3 required balloon angioplasty for either recurrent aortic arch stenosis (n = 1) or right pulmonary stenosis (n = 2) with a mean follow-up time of 3.1 years (IQR 0.5–4.3 years).

**CONCLUSIONS:**

Single-stage repair of IAA-associated APW can be achieved with good survival outcomes in children. However, the need for repeat reintervention or reoperation remains high, and the growth of both the aorta and pulmonary arteries should follow carefully as the patient grows.

**CLINICAL REGISTRATION NUMBER:**

VNCH-RICH-18–96

**Figure:**
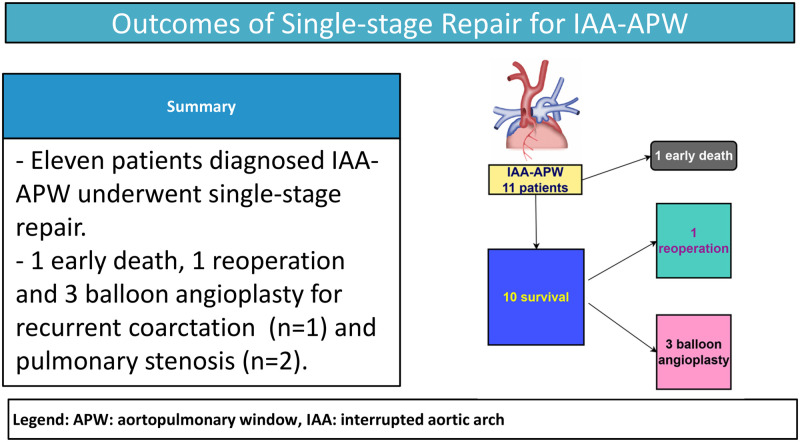

## INTRODUCTION

An aortopulmonary window (APW) associated with an interrupted aortic arch (IAA) as well as a related subgroup [APW, IAA, aortic origin of the right pulmonary artery (RPA) and intact ventricular septum] also known as Berry syndrome, is an extremely rare congenital heart disease (CHD). It accounts for approximately 100 cases reported in the literature [1, 2)]. The lower body perfusion of the patient that is dependent on maintaining a patent ductus arteriosus combined with an unrestricted pulmonary blood flow results in potential haemodynamic instability and possibly cardiogenic shock when the pulmonary vascular resistance drops after birth. This normal postnatal pathophysiologic haemodynamic change explains why some patients reported in the literature required surgical repair during the neonatal period and why, not uncommonly, some patients underwent emergency operations [3–11].

Current outcomes of surgical repair for IAA-associated APW are promising with low mortality and low morbidity [3, 4, 11, 12]. However, in the largest case series from Shanghai Children's Hospital and a multi-institutional study from the Congenital Heart Surgeon Society, mortality and morbidity are still significant, ranging from 15% to 18.8% [5, 6]. In other studies, the postoperative reoperation and reintervention rates are commonly reported, primarily from aortic arch stenosis and RPA stenosis [7–10]. In this study, our goal was to describe the surgical outcomes of single-stage repair for IAA-APW at our institution and review the literature from institutions that have reported more than 4 cases.

## PATIENTS AND METHODS

### Patients

The Vietnam National Children’s Hospital Database was searched for patients admitted who had diagnoses of APW and IAA. The first patient was admitted in September 2012; from September 2012 to December 2021, 11 consecutive patients diagnosed with APW and IAA who underwent surgical correction at our institution were identified.

The hospital ethics committee approved this retrospective study (VNCH-RICH-2021S06) and waived individual informed consent. Data were collected using electronic medical records for surgical procedures and internal cardiology management. Patients were routinely followed up at the outpatient clinic 1 month, 3 months, 6 months and annually after the operation, including telephone checkups.

**Figure 1:**
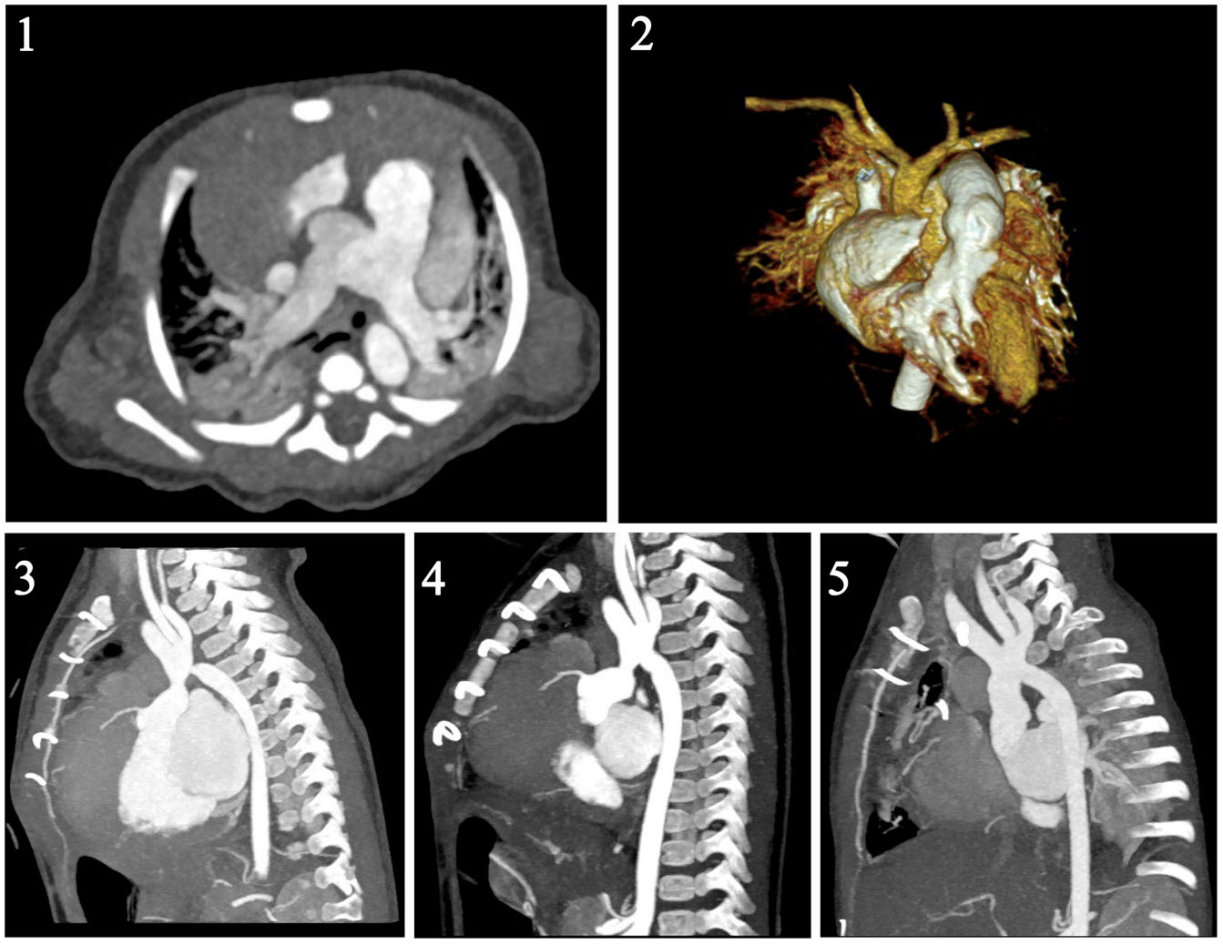
Images of ms-CT of preoperative APW-IAA and postoperative SAS: **(1)** APW type 3; (**2**) 3D-CT reconstruction of IAA type A and APW; (**3**) ms-CT angiogram shows SAS and aortic coarctation after single-stage repair; (**4**) ms-CT angiogram shows residual ascending aorta stenosis after reoperation; (**5**) ms-CT angiogram after the second reoperation shows no residual SAS or coarctation. APW: aortopulmonary window; IAA: interrupted aortic arch; ms-CT: multislice computed tomography; SAS: supravalvar aortic stenosis.

Preoperative diagnosis was performed by transthoracic echocardiography and multislide computed tomography angiography without any catheterization (Fig. 1). The APW was classified according to the Congenital Heart Surgery Nomenclature and Database Project as either type I APW defect close to the RPA or type III complete absence of the aortopulmonary septum [1]. Patients who had a type II APW and an aortic origin of the RPA, combined with IAA, were considered to have Berry’s syndrome [2] and were divided into 2 subtypes: IIa [the continuity with the main pulmonary artery (PA) is still maintained] and IIb [the RPA is haemodynamically and anatomically related with the aorta (Ao)]. The IAA anatomy was described according to the classification of Celoria and Patton [13].

A literature review was performed for APW and IAA utilizing PubMed and Google Scholar search engines. Only studies from institutions reporting more than 4 patients were evaluated for mortality and morbidity after surgical repair related to surgical techniques.

### Surgical techniques

All patients underwent standard cardiopulmonary bypass with hypothermia and a core temperature range from 26°C to 28°C. Bypass was performed using an arterial cannula via a polytetrafluoroethylene shunt connected to the innominate artery or the right common carotid artery (with retrograde right subclavian artery) and bicaval venous cannulation. After bypass was initiated, both PA branches were snared to control the pulmonary blood flow, and the patent ductus arteriosus was kept open to maintain lower-body perfusion. When adequate cardioplegia was introduced into the ascending Ao, aortic arch reconstruction was then initiated with selective cerebral perfusion. Then, a direct end-to-side anastomosis was performed between the descending Ao and the aortic arch when the superior rim of the APW was lower than the proximal portion of the aortic arch by at least 5–7 mm. In cases in which the resection of the ductal tissue resulted in a considerable distance between the aortic arch and the descending Ao, an additional patch using the autologous tissue of the sacrificed left subclavian artery (IAA type B) or a bovine pericardial patch (CardioCel, LeMaitre Cascular, Burlington, MA, USA) was added to provide a tension-free anastomosis at the anteroinferior border of the aortic arch repair.

After the aortic arch reconstruction, the APW was closed during the rewarming phase. The APW was accessed through the anterior wall in 3 patients: the APW was closed using an intra-aortic baffle of bovine pericardial patch in 2 patients (APW types IIb and III), and the APW was closed directly (APW type IIa) in 1 patient. Five patients went through the RPA detachment technique. The RPA and main PA with the left pulmonary artery were further dissected out to the hilum for a tension-free anastomosis before reconnection. The aortic defect was repaired by a bovine pericardial patch. In 2 patients, the RPA was translocated anterior to the Ao (Lecompte manoeuvre) using an autologous pericardial conduit of 9 mm. In the remaining 3 patients, the posterior RPA wall was kept reconnected to the main PA, and the anterior wall was patched with an autologous pericardial patch.

**Figure 2:**
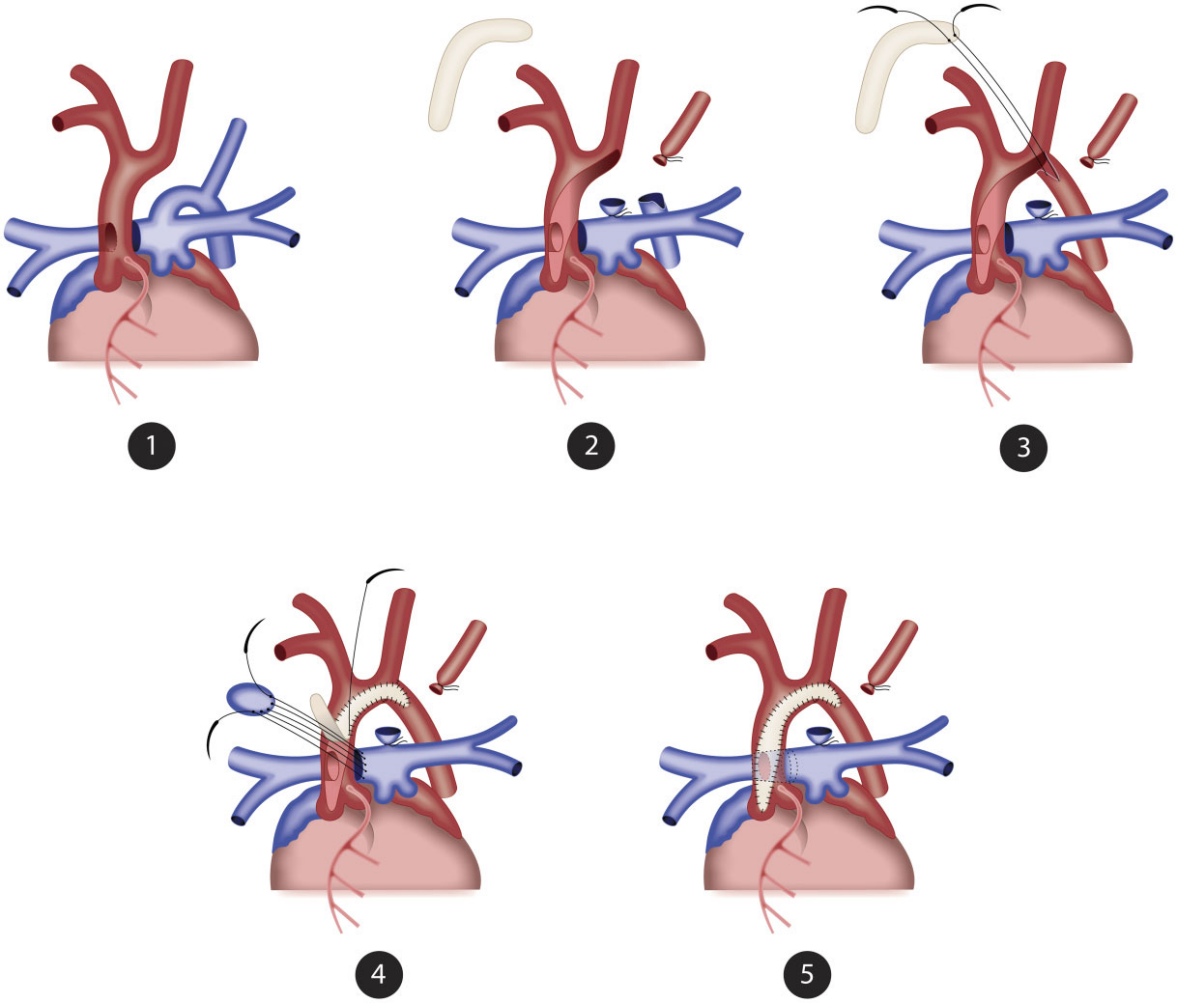
Surgical technique of single-stage repair using intra-aortic baffle combined with a boomerang patch for aortic reconstruction: (**1**) Anatomic lesion of IAA-APW with anomalous origin of the right pulmonary artery. (**2**) The PDA was removed, and the entire Ao was open from the aortic arch through the ascending Ao up to the noncoronary sinus. (**3**) A boomerang bovine pericardial patch was used to enlarge the aortic arch up to the ascending Ao. (**4**) A trimmed bovine pericardial patch was used to direct the blood from the main PA to the RPA. (**5**) The boomerang patch is used to expand the ascending Ao. Ao: aorta; APW: aortopulmonary window; IAA: interrupted aortic arch; PDA: patent ductus arteriosus; RPA: right pulmonary artery.

The 3 most recent cases with the RPA originating from the ascending Ao, who underwent operations in 2021, were approached using a novel technique developed by our institution. After selective cerebral perfusion, an incision was made at the lateral border of the ascending Ao opposing the APW, crossing superior to the aortic arch and inferior to the noncoronary sinus. The posterior wall of the descending Ao was then reconnected to the aortic arch using an 8.0 polypropylene suture (Corolene, Péters Surgical, Inc., Bobigny, Ile-de-France, France), and the anterior wall of the descending Ao was incised. A boomerang bovine pericardial patch was sewed from the descending Ao through the aortic arch to complete the aortic arch reconstruction (Video 1). The APW was easily approached and closed with a bovine pericardial patch through the ascending Ao with visible RPA origin and left coronary ostium (Fig. 2). Subsequently, the boomerang pericardial patch was extended to enlarge the ascending Ao up to the coronary sinus to avoid supravalvar aortic stenosis (SAS).

**Figure 3:**
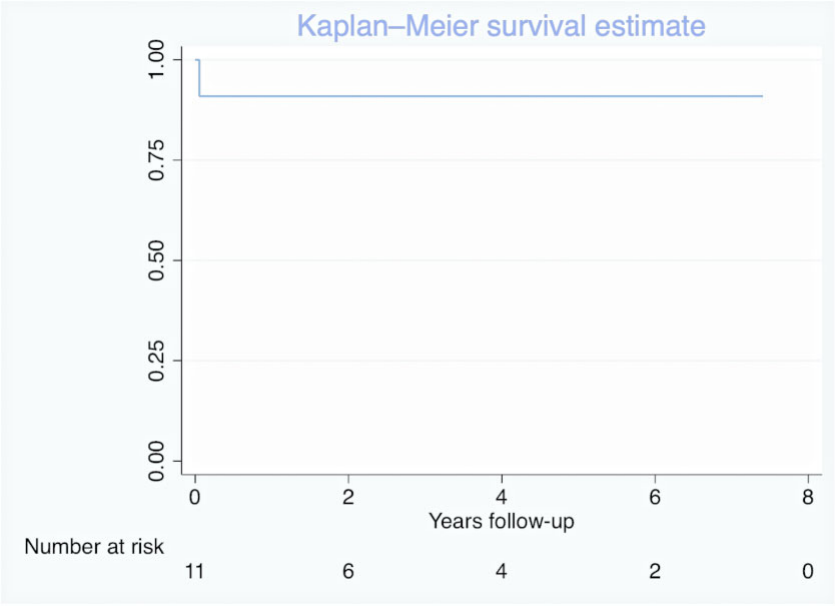
Kaplan–Meier survival of patient with IAA-APW. IAA-APW: interrupted aortic arch- aortopulmonary window.

### Statistical analysis

Continuous variables are expressed as the mean with standard deviation or the median with a range for skewness variables. Categorical variables are presented as frequency and percentages. Data were analysed with Stata software, version 17.0 (Stata Corp LLC, College Station, TX, USA). Freedom from events (reoperation) was estimated using the Kaplan–Meier method.

## RESULTS

Between 2012 and 2021, a total of 11 consecutive patients (5 boys and 6 girls) underwent single-stage surgical repair for APW and IAA at our institution. The median age at operation was 27 [interquartile range (IQR) 6–79] days, and the median weight at operation was 3.5 (IQR 2.8–4.5) kg. Two patients (18%) were mildly premature (35 and 36  gestational weeks) and required prostaglandin E1 infusion to keep the ductus patent. Both underwent surgery at 38.5 gestational weeks. No infant had a prenatal diagnosis at presentation. None of the patients had DiGeorge syndrome, which was confirmed by the fluorescence in situ hybridization test.

Three patients (27%) required preoperative ventilation; 3 (27%) were admitted to the hospital with cardiogenic shock; 7 (63.6%) needed inotropic support; and 5 (45.4%) underwent an emergency operation upon diagnosis. One patient had acute kidney injury following cardiogenic shock when admitted to our hospital. No liver failure was detected upon the admission of patients in this series. Moreover, 9 (81.8%) patients had IAA type A and 2 (18%) had IAA type B. The APW morphology was type I in 1 (9%) patient, type IIa in 1 (9%), type IIb in 3 (27%) and type III in 6 (54.5%). In addition, 8 (81.8%) patients had RPA from the ascending Ao, with 6 (54.5%) having RPA originating exclusively from the lateral side of the ascending Ao. Patient demographics and surgical technique are described in Table 1.

**Table 1:** Patient demographics and outcomes

Patient	Age	Weight, g	Preoperative features	Pre-op stay/ventilation time, days	IAA type	APW type	Arch repair technique	APW technique	Bypass time, min	ACC time, min	ACP time, (min)	ICU stay, days	Hospital stay, days	Reoperation/reintervention/complication
1	26 Days	2.8	Cardiogenic shock	20	A	III	Direct	Intra-Ao baffle	162	82	45	10	30	Died/ECMO
2	11 Months	6.5	Congestive heart failure, PDA open	2	A	II (IIb)	Direct	Intra-Ao baffle	122	90	25	3	17	None
3	2.5 Months	4.6	Congestive heart failure, ductus closing, PGE1 infusion	8	A	II (IIb)	Direct	RPA detachment	146	114	62	3	18	None
4	3 Days	3.0	Congestive heart failure, ductus closing, PGE1 infusion	2	A	I	Direct	RPA detachment	121	81	50	19	32	None
5	4 Months	3.2	Congestive heart failure, PDA open	24	B	III	Direct	RPA detachment (Lecompte)	126	80	20	6	37	None
6	2.5 Months	3.7	Congestive heart failure, PDA open	8	A	III	Direct	RPA detachment	171	82	39	5	21	None
7	19 Days	2.5	Congestive heart failure, ductus closing, PGE1 infusion	6	A	II (IIa)	Direct	Intra-Ao baffle	69	40	15	7	20	Balloon angioplasty/aortic arch stenosis
8	1.5 Months	4.5	Cardiogenic shock, preoperative ventilation, inotropic infusion, PGE1 infusion	3/3	A	III	Patch	RPA detachment (Lecompte)	122	64	40	12	19	Reoperation/Supra-aortic stenosis
9	9 Days	4.1	Cardiogenic shock, preoperative ventilation, inotropic infusion, PGE1 infusion	1/1	B	III	Patch	Intra-Ao baffle	134	88	45	6	27	Balloon angioplasty/RPA stenosis
10	26 Days	2.8	Congestive heart failure, ductus closing, PGE1 infusion	21	A	II (IIb)	Patch	Intra-Ao baffle	93	84	40	4	32	None
11	4 Days	3.5	Cardiogenic shock, preoperative ventilation, inotropic infusion, PGE1 infusion	1/1	A	III	Patch	Intra-Ao baffle	108	60	40	4	20	Balloon angioplasty/RPA stenosis

ACC: aortic cross-clamp; ACP: anterior cerebral perfusion; Ao: aorta; APW: aortopulmonary window; ECMO: extracorporeal membrane oxygenation; IAA: interrupted aortic arch; ICU: intensive care unit; PDA: patent ductus arteriosus; PGE1: prostaglandin E1; RPA: right pulmonary artery.

Three (27%) patients had associated cardiac anomalies including atrial septal defect. The mean cardiopulmonary bypass time, aortic cross-clamp time and selective cerebral perfusion time were 122 (IQR 108–146) min, 82 (IQR 64–88) min and 40 (IQR 25–45) min, respectively. Seven patients (63.6%) had an extended end-to-side anastomosis, and the remaining 4 patients had an additional patch augmentation for IAA repair. The RPA detachment technique was applied for APW closure in 5 (45.4%) patients; 3 (27%) underwent a simple intra-aortic baffle for APW closure; and 3 (27%) underwent closure of the APW by an intra-aortic baffle combined with ascending aortic enlargement by a boomerang patch.

### Early postoperative outcomes

One perioperative death (9%) occurred in 2012; it was the first patient in our series. She was admitted to the hospital at 6 days old with cardiogenic shock due to a closed ductus, and an infusion of prostaglandin E1 was given for preoperative patient stabilization. She underwent single-stage repair at age 26 days. However, on postoperative day 2, sepsis shock occurred due to nosocomial infection with multiorgan failure. She died on postoperative day 10, despite extracorporeal membrane oxygenation support. No patients were lost to follow-up, and the median follow-up time was 2.1 (IQR 0.6–4.6) years. No late deaths occurred during follow-up at either 1 year or 5 years (Figure 3).

The median time of postoperative ventilation was 67.3 (IQR 38–111) h, and their median stay in the intensive care unit was 5.6 (IQR 3.9–9.8) days. Four patients (36%) had severe PA hypertension determined by echocardiography, and they required treatment by pulmonary vasodilator postoperatively with an iloprost infusion (0.5–2 ng/kg/min), followed by oral intake of sildenafil (0.3 mg–6/kg/dose, 3–4 times a day). Six patients (54.5%) had low cardiac output syndrome, and 4 (36%) had capillary leakage syndrome. One patient developed a wound infection and 1 patient had pneumonia. None of the patients in our series suffered left vocal cord paralysis.

### Early morbidity

One patient (patient 8), who presented with preoperative cardiogenic shock and a low ejection fraction (41%), required 2 reoperations after the initial repair. The patient had SAS with an echocardiographic peak gradient (PG) max of 57 mmHg at 6 months postoperatively. Reoperation was indicated to release the residual stenosis. However, ascending aortic stenosis still developed after the first reoperation with an echocardiographic PG max of 47 mmHg in association with left ventricular (LV) dilatation and LV dysfunction with an LV ejection fraction that ranged from 40% to 45%, combined with severe mitral valve regurgitation. The second reoperation was performed 3 months after the first reoperation, which included RPA detachment and placement of a generous bovine pericardial patch to enlarge the narrowed Ao combined with mitral annuloplasty. The most current follow-up echocardiography demonstrated a PG max across the ascending Ao of 16 mmHg, mild mitral valve regurgitation with an LV ejection fraction of 40% and a residual origin of RPA stenosis with a PG max of 42 mmHg. This patient is on the waiting list for reintervention of balloon angioplasty for RPA.

Another patient (patient 7) had discrete aortic arch stenosis at the anastomotic site with a PG max of 82 mmHg 2 months postoperatively. He was operated on during the neonatal period; his body weight at operation was 2.5 kg, and a direct anastomosis between the aortic arch and the descending Ao was performed without patch augmentation. He underwent successful balloon angioplasty to relieve the obstruction, and the latest follow-up echocardiogram showed no residual stenosis with a PG max of 14 mmHg across the aortic isthmus.

Two more patients (patients 9 and 11) underwent successful RPA balloon angioplasty for RPA obstruction (the position of the window patch and the PG max through the stenotic site were 43 mmHg and 45 mmHg, respectively) at 3 and 5 months postoperatively, respectively. Both patients showed no echocardiographic evidence of restenosis at their latest follow-ups.

In this study, the functional status of the patients at the final follow-up contact showed that all patients but 1 had no symptoms of heart failure and had adequate somatic growth. The 1 exception was a patient with cardiogenic shock and low ejection fraction preoperatively who had a functional status of Ross class 3.

The literature review revealed several case series with more than 4 patients; these are listed in Table 2.

**Table 2:** Literature review of series with >5 patients

Authors	n	Early death	Late death	Follow-up time (year)	Arch reoperation intervention	RPA reintervention	Supravalvar stenosis
McElhinney et al.^7^ (1998)	8	2 (APW2; IAA B); (APW1; IAA A)	0	10.5	2(3) (APW1; IAA A) (APW2; IAA B)	1 (APW2; IAA B)	
Hew et al.^21^ (2001)	7	0	0	6.6	1	1	
Bagtharia et al.^13^ (2004)	6	u	u	u	u	u	
Konstantinov et al.^6^ (2005)	20	2	1		8	5	
Konstantinov et al.^11^(2010)	5	0	0	17.6	1 (APW1; IAA B)	0	
Barnes et al.^12^ (2011)	9	0	0	u	u	u	
Murin et al.^4^ (2012)	8	1	0	9.8	0	0	
Roubertie et al.^3^ (2015)	11	0	0	6	0	0	
Alsoufi et al.^8^ (2016)	6	0	0	8.2	3	1	2
Hu et al.^5^ (2017)	16	2 (APW2; IAA A)	1 (APW2; IAA B)	4.8	1 (APW2; IAA A)	3 (APW2; IAA A) (APW3; IAA A) (APW2; IAA B)	
This study (2021)	11	1 (APW3; IAA A)	0	3	1 (APW1; IAA A)	3	1
**Total**	94 (84)				17 (20.2%)		

APW: aortopulmonary window; IAA: interrupted aortic arch.

## DISCUSSION

### Mortality

Although some recent reports have shown no early or late mortality (3, 4), patient death is not uncommon due to the complexity of the surgical repair or late presentation with cardiogenic shock [5–7]. The associated left-sided obstruction in patients diagnosed with APW is considered to increase the risk of mortality and adverse outcomes [6, 14].

Despite some recent reports about the prenatal diagnosis of IAA-associated APW [15–17], most of the patients described in the literature were diagnosed when they were admitted to the hospital, including those in our study [3–5, 7, 14]. Except for the first patient, who died of septic shock postoperatively, we have had no recent deaths among the remaining patients in our series despite the fact that we have many late presentations, which makes the management more challenging, including those who underwent emergency operations with a constrictive ductus arteriosus. Nonetheless, the number of deaths associated with this complex CHD should continue to improve with early diagnosis in neonates, especially with prenatal echocardiography.

### Aortic arch reintervention or reoperation

In a previous study, IAA type B was identified as an incremental risk factor for arch reintervention after repair of APW and IAA [6]. The finding is correlated with the less frequent use of additional patches for aortic arch reconstruction in this multicentre analysis. However, we believe that additional patch aortoplasty is not always necessary in all patients with IAA. In our study, 7 (63.6%) patients underwent direct extended end-to-side anastomosis without the patch, with the smallest patient (2.5 kg) requiring aortic arch reintervention (balloon angioplasty). Murin *et al.* [4] studied 3 patients (42%) who underwent arch reconstruction by direct end-to-side anastomosis and 1 patient with an extended end-to-end anastomosis in addition to a left subclavian flap plasty, and none of these patients developed aortic arch obstruction during the follow-up period. In our experience, patients present with a well-developed aortic arch, and the ductal tissue does not exceed too far into the descending Ao owing to a low risk of obstruction. We suspect that careful evaluation of the geometry of the patient’s aortic arch and intraoperative anatomy will help determine the appropriate surgical technique: a direct anastomosis or an additional patch repair.

The Congenital Heart Surgeons Society multi-institution study found that those who had aortic arch repair by a method other than direct anastomosis with patch augmentation are more likely to require reintervention [18]. Aortic arch reconstruction with an additional patch appears to be the best solution for IAA repair, as proved by recent studies, because no patients required arch reintervention during follow-up [3, 5]. We recently adopted a new surgical technique for aortic arch reconstruction with a boomerang bovine pericardial patch (CardioCel) to avoid circumferential isthmus narrowing and help enlarge the ascending arch. In our experience with aortic arch reconstruction, an anteroposterior patch to enlarge the aortic arch will help avoid a gothic shape aortic arch geometry and avoid left bronchus compression (Central Image).

### Supravalvular aortic stenosis

In the literature, SAS is not frequently seen during postoperative follow-up for this complex lesion, but this complication usually requires reoperation and is a complex redo procedure that is related primarily to the origin of RPA and the combination with RPA stenosis. Alsoufi *et al.* reported 2 cases with SAS: 1 patient required 1 reoperation and another patient underwent 2 reoperations for recurrent obstruction at the ascending Ao [8]. Moreover, Uematsu *et al.* described a patient who suffered progressive LV failure 7 months after neonatal surgical repair [19]. The authors stated that this is a fatal complication and recommended that division of the Ao and PA is indispensable for neonates or small infants. Some authors also consider that the intra-aortic baffle technique may have 2 weaknesses: growth potential and size of the patch [20, 21]. Hu *et al.* recommended trimming the intra-aortic patch precisely to avoid SAS and RPA stenosis, but in their series, no patient developed SAS during follow-up [5]. The mechanism of SAS occurrence remains unclear, but the patch was taken into the lumen of the ascending Ao in patients who underwent intra-aortic baffle repair.

In our series, 1 patient underwent intracardiac baffle repair for APW and suffered SAS. After that case, we applied a new surgical technique using the intra-aortic baffle combined with a single patch enlargement for the ascending Ao and the entire aortic arch to potentially avoid this complication. Our new surgical technique has many advantages: The approach for APW is straightforward with a clear surgical vision of the border of the APW. The suture line for the APW repair is laying on the lumen of the vessels with less risk of bleeding, thereby maintaining the posterior wall of the RPA, easily avoiding damage to the coronary ostium and potentially avoiding SAS by enlarging the entire Ao including the aortic arch. However, close follow-up is mandatory for patients with APW-IAA repair to maintain optimal outcomes following this complex procedure.

### RPA stenosis

In the literature, the most common complication for all patients with APW-IAA is RPA stenosis [5–8, 22]. The suspected mechanism of postoperative RPA stenosis in patients in whom an intra-aortic baffle was utilized is that the patch may shift towards the PA, causing RPA stenosis and limiting potential RPA growth. In our series, 2 patients developed RPA stenosis and required successful RPA balloon angioplasty soon after the operation during follow-up. In these patients, the intra-aortic baffle repair was applied. The other 3 patients in our study also had the intra-aortic baffle technique and did not develop RPA stenosis, with 2 of the 3 patients being neonates weighing 2.5 kg and 2.8 kg, respectively. Recently, Shi *et al.* reported their results of the intra-cardiac baffle technique for IAA type B and APW type III in neonatal repair due to a high rate of reoperation needed for RPA stenosis and a potential problem of the SAS [9].

Moreover, RPA stenosis is also seen with other surgical techniques. Hu *et al.* recommended that the aortic cuff technique should not be the procedure of choice in this complex CHD lesion due to a higher reintervention rate with RPA obstruction. A total of 3/5 patients in their study and 2/6 patients in another study accounted for 45% of patients who had this aortic cuff technique who developed RPA stenosis during follow-up [5, 8, 23]. With a shortening of the ascending Ao after harvesting the aortic cuff, the RPA is compressed, which is the main cause of RPA stenosis. Another technique utilizing RPA detachment has a promising outcome with no reoperations or reinterventions for RPA obstruction [5, 24]. In our series, 5 patients who underwent RPA detachment also showed no evidence of RPA obstruction during follow-up. On the contrary, the RPA detachment technique interrupts the native connection between the RPA and left pulmonary artery, which may limit RPA growth and may be technically demanding for RAP reimplantation to avoid a complication such as myocardial ischaemia, as described by Duyen *et al.* [25].

### Limitations

This study has several limitations. Although IAA-APW is a very rare CHD, the number of patients in this study is relatively small with a relatively short mean follow-up time. Even after an “optimal” repair, the possibility of obstruction of the great artery as the child grows cannot be ignored, and close and careful follow-up for this possibility is needed. Furthermore, large outcome studies for indications for each surgical technique chosen for aortic arch reconstruction and APW closure are lacking, and the results are potentially affected by surgical selection bias.

## CONCLUSIONS

Primary repair of IAA-associated APW can be performed with good outcomes even in low- to middle-income countries such as Vietnam. RPA detachment is a promising technique with a low reintervention or reoperation rate for RPA stenosis. However, rates of complications such as SAS, aortic arch obstruction or RPA stenosis remain high after surgical repair of this complex malformation. Close follow-up is mandatory for these patients for long-term optimal outcomes.

## Data Availability

All relevant data are within the manuscript and its supporting information files. The authors confirm that the data supporting the findings of this study are available from the corresponding author on request.

## Abbreviations

Ao: Aorta
APW: Aortopulmonary window
CHD: Congenital heart disease
IAA: Interrupted aortic arch
LV: Left ventricular
PA: Pulmonary artery
PG: Peak gradient
RPA: Right pulmonary artery
SAS: Supravalvar aortic stenosis

## References

[1] Jacobs JP, Quintessenza JA, Gaynor JW, Burke RP, Mavroudis C. Congenital Heart Surgery Nomenclature and Database Project: aortopulmonary window. Ann Thorac Surg 2000;Mar69:44–9. 10.1016/s0003-4975(99)01236-9 10798415

[2] Berry TE, Bharati S, Muster AJ, Idriss FS, Santucci B, Lev M et al Distal aortopulmonary septal defect, aortic origin of the right pulmonary artery, intact ventricular septum, patent ductus arteriosus and hypoplasia of the aortic isthmus: newly recognized syndrome. Am J Cardiol 1982;Jan49:108–16. 7053598 10.1016/0002-9149(82)90284-3

[3] Roubertie F, Kalfa D, Vergnat M, Ly M, Lambert V, Belli E. Aortopulmonary Window and the Interrupted Aortic Arch: Midterm Results With Use of the Single-Patch Technique. Ann Thorac Surg 2015;Jan99:186–91. 25440264 10.1016/j.athoracsur.2014.08.023

[4] Murin P, Sinzobahamvya N, Blaschczok H, Photiadis J, Haun C, Asfour B et al Aortopulmonary Window Associated with Interrupted Aortic Arch: Report of Surgical Repair of Eight Cases and Review of Literature. Thorac Cardiovasc Surg 2012;Apr60:215–20. 22252330 10.1055/s-0031-1298061

[5] Hu R, Zhang W, Liu X, Dong W, Zhu H, Zhang H. Current outcomes of one-stage surgical correction for Berry syndrome. J Thorac Cardiovasc Surg 2017 ;153:1139–47. 28089641 10.1016/j.jtcvs.2016.11.058

[6] Konstantinov IE, Karamlou T, Williams WG, Quaegebeur JM, del Nido PJ, Spray TL, Congenital Heart Surgeons Society et al Surgical management of aortopulmonary window associated with interrupted aortic arch: Congenital Heart Surgeons Society study. J Thorac Cardiovasc Surg 2006;131:1136–41.e2. 16678601 10.1016/j.jtcvs.2005.03.051

[7] McElhinney DB, Reddy VM, Tworetzky W, Silverman NH, Hanley FL. Early and Late Results After Repair of Aortopulmonary Septal Defect and Associated Anomalies in Infants < 6 Months of Age. Am J Cardiol 1998;Jan81:195–201. 9591904 10.1016/s0002-9149(97)00881-3

[8] Alsoufi B, Schlosser B, McCracken C, Kogon B, Kanter K, Border W et al Current Outcomes of Surgical Management of Aortopulmonary Window and Associated Cardiac Lesions. Ann Thorac Surg 2016;Aug102:608–14. 27207392 10.1016/j.athoracsur.2016.02.035

[9] Shi XC, Weng JB, Yu J, Ma XH, Pu YQ, Ying LY et al Outcomes of One-Stage Surgical Repair for Berry Syndrome in Neonates. Front Cardiovasc Med 2021;Jan 268:790303. 35155602 10.3389/fcvm.2021.790303 PMC8825814

[10] Backer CL, Mavroudis C. Surgical management of aortopulmonary window: a 40-year experience. Eur J Cardiothorac Surg 2002;21:773–9 10.1016/s1010-7940(02)00056-8 12062263

[11] Konstantinov IE, Oka N, d'Udekem Y, Brizard CP. Surgical repair of aortopulmonary window associated with interrupted aortic arch: Long-term outcomes. J Thorac Cardiovasc Surg 2010;Aug140:483–4. 20363477 10.1016/j.jtcvs.2009.12.043

[12] Barnes ME, Mitchell ME, Tweddell JS. Aortopulmonary Window. Semin Thorac Cardiovasc Surg Pediatr Card Surg Annu 2011;Jan14:67–74. 21444051 10.1053/j.pcsu.2011.01.017

[13] Celoria C, Absence of the arch of the aorta is a ‘rare anomaly. A careful search of the 7.

[14] Bagtharia R, Trivedi KR, Burkhart HM, Williams WG, Freedom RM, Van Arsdell GS et al Outcomes for patients with an aortopulmonary window, and the impact of associated cardiovascular lesions. Cardiol Young 2004;Oct14:473–80. 15680067 10.1017/S1047951104005025

[15] Ghelani SJ, Quinonez LG, Rathod RH. Prenatal Diagnosis and Management of Berry Syndrome, a Rare Conotruncal Anatomy. Circulation 2015;Oct 20132:1593–4. 26481566 10.1161/CIRCULATIONAHA.115.017366

[16] Zhang X, Liu XW, Gu XY, Han JC, Hao XY, Fu YW et al Prenatal diagnosis of Berry syndrome by fetal echocardiography: A report of four cases. Echocardiography 2018;Apr35:563–5. 29430703 10.1111/echo.13832

[17] Matsubara Y, Ota M, Bito A, Katayama T, Matsubara K, Ito M. Prenatal diagnosis of Berry syndrome by fetal echocardiography. Ultrasound Obstet Gynecol 2010;Mar35:374–6. 20104532 10.1002/uog.7565

[18] McCrindle BW, Tchervenkov CI, Konstantinov IE, Williams WG, Neirotti RA, Jacobs ML, Congenital Heart Surgeons Society et al Risk factors associated with mortality and interventions in 472 neonates with interrupted aortic arch: A Congenital Heart Surgeons Society study. J Thorac Cardiovasc Surg 2005;Feb129:343–50. 15678045 10.1016/j.jtcvs.2004.10.004

[19] M Uematsu MU, Severe ascending aortic stenosis after one-stage repair of aortopulmonary window and interrupted aortic arch. Available from https://pubmed.ncbi.nlm.nih.gov/7963844/7963844

[20] Codispoti M, Mankad PS. One-stage repair of interrupted aortic arch, aortopulmonary window, and anomalous origin of right pulmonary artery with autologous tissues. Ann Thorac Surg 1998;Jul66:264–7. 9692484 10.1016/s0003-4975(98)00364-6

[21] Burke RP, Rosenfeld HM. Primary repair of aortopulmonary septal defect interrupted aortic arch, and anomalous origin of the right pulmonary artery. Ann Thorac Surg 1994;Aug58:543–5. 8067861 10.1016/0003-4975(94)92250-0

[22] Hew CC, Bacha EA, Zurakowski D, del Nido PJ, Mayer JE, Jonas RA. Optimal surgical approach for repair of aortopulmonary window. Cardiol Young 2001;Jul11:385–90. 11558947 10.1017/s104795110100049x

[23] Park SY, Joo HC, Youn YN, Park YH, Park HK. Berry Syndrome Two Cases of Successful Surgical Repair: Two Cases of Successful Surgical Repair. Circ J 2008;72:492–5. 18296853 10.1253/circj.72.492

[24] Chang YH, Sung SC, Kim H, Lee HD. Anterior Translocation of the Right Pulmonary Artery for Relief of Airway Compression in the Repair of Distal Aortopulmonary Window and Interrupted Aortic Arch. Ann Thorac Surg 2012;Jun93:e159–61–e161. 22632536 10.1016/j.athoracsur.2011.12.027

[25] Duyen MD, Haranal MY, Dillon J, Sivalingam S. A rare complication of myocardial ischaemia following single-stage repair in a case of Berry syndrome. Interact CardioVasc Thorac Surg 2020;Oct 131:576–7. 10.1093/icvts/ivaa126 32772077

